# Knowledge Gaps Regarding Alcohol Consumption During Pregnancy and Its Effect on the Fetus: A Systematic Review Focused on Women

**DOI:** 10.3390/jcm14197047

**Published:** 2025-10-05

**Authors:** María Botella-López, María-Teresa Cortés-Tomás

**Affiliations:** Department of Basic Psychology, Faculty of Psychology, University of Valencia, 46010 Valencia, Spain; maria.t.cortes@uv.es

**Keywords:** alcohol, pregnancy, fetal alcohol spectrum disorder, FASD, knowledge, risk, pregnant women, women of childbearing age

## Abstract

**Background/objectives:** Alcohol use during pregnancy can result in adverse outcomes for the offspring, including Fetal Alcohol Spectrum Disorders (FASD). Psychosocial and contextual factors may influence gestational alcohol intake and women’s risk perception. This systematic review aimed to assess pregnant women’s and women of childbearing age’s perceived risk of alcohol use during pregnancy and to evaluate their knowledge of its potential effects on children. **Methods:** Following the PRISMA guidelines, a systematic search was conducted in Web of Science, PubMed and PsycArticles databases for studies published up to May 2025. Eligible studies examined gestational alcohol use, risk perception, or knowledge of fetal consequences among pregnant women or women of reproductive age. Methodological quality was assessed with the Critical Appraisal of Qualitative Studies tool from the Centre for Evidence-Based Medicine (CEBM). **Results:** Twenty-nine studies were included. Reported prevalence of alcohol consumption during pregnancy varied considerably across settings. A substantial proportion of women perceived alcohol use during pregnancy as acceptable, often depending on quantity, frequency, type of beverage, or stage of gestation. Knowledge of FASDs was generally limited and frequently restricted to physical malformations. Misconceptions were more common among women with prior alcohol use. The findings highlight persistent gaps in risk perception and knowledge about FASDs. **Conclusions:** Prevention strategies should not be limited to pregnant women but should also target women of childbearing age, especially those with active drinking patterns, as well as their immediate sociocultural environment. Strengthening professional training, community-based interventions, and consistent public health messaging are essential to reduce gestational alcohol exposure.

## 1. Introduction

Alcohol consumption during pregnancy is the leading non-genetic cause of intellectual disability in offspring [1]. In 1973, Lemoine and his colleagues coined the term Fetal Alcohol Syndrome (FAS) to describe the effects on the fetus of maternal alcohol consumption during pregnancy, characterised by a specific facial phenotype, prenatal or postnatal growth deficiencies and damage to the central nervous system [2]. However, more recent research has shown that in the total or partial absence of the typical facial characteristics of FAS, neurological and behavioural impairments were also observed in children whose mothers had consumed different quantities of alcohol during pregnancy [3,4]. To accommodate all conditions resulting from prenatal exposure to alcohol, the term Fetal Alcohol Spectrum Disorders (FASD) was defined, which includes both FAS (considered the manifestation with most symptoms) and partial FAS (pFAS), Alcohol-Related Neurodevelopmental Disorder (ARND) and Alcohol-Related Congenital Malformations (ARCM) [5,6,7].

Although the teratogenic effects of alcohol are supported by the scientific community [8,9,10], there is still no agreement on the minimum amount of alcohol necessary to produce adverse effects on the fetus, so the current consensus advocates total abstinence from alcohol during pregnancy as the safest measure to prevent FASDs [11].

Despite this warning, a large number of pregnant women still drink alcohol. A study by Popova et al. (2017) found that the overall prevalence of alcohol consumption during pregnancy was 9.8% [12]. However, there are significant geographical differences. Europe shows the highest prevalence (25.2%), followed by the Americas (11.2%) and Africa (10.0%). In Asia, the prevalence is considerably lower, with 1.8% in South-East Asia and 8.6% in the Western Pacific. Similar patterns are observed for FAS, with Europe presenting the highest rate (37.4 cases per 10,000), compared with 16.6 in the Americas, 14.8 in Africa, 12.7 in the Western Pacific, and 2.7 in South-East Asia. These variations reflect the influence of cultural, socioeconomic, and healthcare factors on drinking patterns. In Spain, it is estimated that 10–15% of pregnant women consume alcohol during pregnancy; however, when objective biomarkers such as maternal hair analysis are applied, prevalence rises to 64.7%, highlighting the substantial underreporting of alcohol use [13].

One of the most important predictors of alcohol intake during pregnancy is the pattern of alcohol consumption prior to pregnancy [14,15,16]. According to the latest Survey on Alcohol and Drugs in Spain (EDADES, 2024), around 74% of women aged between 15 to 44 have consumed alcohol in the last year, and approximately 6 out of 10 women in this age group have consumed alcohol in the last month [17]. In terms of risky consumption patterns, such as acute alcohol intoxication (more commonly known as drunkenness), 16.2% of women aged between 15 and 44 have consumed alcohol in this way in the last 12 months. Regard to binge drinking (BD), defined as the consumption of 5 or more alcoholic drinks for men or 4 or more drinks for women on the same occasion (consecutively or within a two-hour interval), approximately 12% of women in this age group have engaged in BD in the last month.

These data reveal quite alarming numbers regarding the drinking patterns of women of reproductive age. On the one hand, there is the risk of drinking alcohol during unplanned or unwanted pregnancies; on the other hand, we must not forget the time lag between conception and recognition of pregnancy (which in some cases can be weeks), which increases the risk of alcohol consumption during pregnancy and the possible teratogenic effects of this substance on the offspring.

This reality highlights the importance of understanding the factors associated with alcohol consumption during pregnancy. In addition to previous drinking patterns, the influence of other sociodemographic factors on alcohol intake during pregnancy has also been extensively researched. These factors include age [18,19,20,21], educational level [18,19,20,22], nulliparity/multiparity [18,23], and tobacco use [18,19,24]. Nevertheless, research on the psychosocial factors that might also impact the choice to drink alcohol during pregnancy is limited [16,25]. Among these factors, the attitudes and beliefs of pregnant women or women of childbearing age regarding the risks of drinking alcohol during pregnancy are particularly notable, as is their awareness of the teratogenic effects that consuming any amount of alcohol during this period can have on the fetus.

To address the discrepancies between these factors, this systematic review has two objectives: (a) to evaluate the perceived risks associated with alcohol consumption during pregnancy among pregnant women or women of childbearing age, and (b) to assess their knowledge of the potential impact of alcohol on fetal development.

## 2. Materials and Methods

This systematic review was conducted in accordance with the PRISMA (Preferred Reporting Items for Systematic Reviews and Meta-analyses) guidelines [26].

### 2.1. Search Strategy

A systematic review of studies was conducted using the Web of Science (WOS), PubMed and PsycArticles databases.

The search was conducted between February and May 2025. A preliminary analysis was performed to identify specific keywords for each database related to FASDs/SAF and gestational alcohol exposure; attitudes, knowledge and risk perception; and pregnancy and women of childbearing age, using the Boolean operators AND/OR. The WOS database search was conducted in the Topic field (title, abstract and keywords); in Pubmed, the All fields field was used; and in Psycarticles, the search was conducted in any field except full text *(NOFT)*.

In the WOS database, the terms fetal alcohol syndrome OR FAS OR fetal alcohol exposure OR fetal alcohol spectrum disorders OR FASD OR fetal alcohol spectrum OR prenatal alcohol exposure OR PAE OR Neonatal ethanol exposure OR neurobehavioral disorder associated with prenatal alcohol exposure OR foetal alcohol syndrome OR Alcohol-exposed pregnancy OR fetal alcohol effects OR prenatal maternal alcohol exposure AND knowledge OR beliefs OR pregnancy warning label OR risk perception OR attitudes OR awareness OR perceived alcohol safety OR reasons OR thoughts OR educational resources AND pregnancy, pregnant patient OR pregnant women OR expectant mother OR mother OR multiparous women OR primiparous women were combined.

In PubMed, the above terms were combined and the following were added: AEP OR foetal alcohol spectrum disorders OR neurobehavioral deficits associated with prenatal alcohol exposure OR alcohol prenatally OR intrauterine alcohol exposure OR fetal alcohol syndrome disorder AND FASD prevention messaging OR pregnancy alcohol risk perception OR perceptions OR health message perceptions AND pregnant mothers OR pregnancy exposure OR new mothers OR postpartum women OR women of childbearing age.

Finally, in the PsycArticles database, all of the above terms were combined with: FAE OR alcohol exposed children OR neuropsychological deficits associated with heavy prenatal alcohol exposure OR prenatal exposure to alcohol OR alcohol prenatally OR alcohol induced behavioral alterations OR effects of alcohol exposure OR alcohol induced deficits OR gestational alcohol exposure OR prenatal ethanol exposure OR prenatal exposure to ethanol OR neonatal alcohol exposure OR effects of neonatal alcohol AND health risks OR judgment AND birth mother OR gestation OR motherhood.

### 2.2. Selection/Exclusion Criteria

Articles were included in the review if (a) they were written in English or Spanish, (b) they included pregnant women and/or women of reproductive age as the study population, and (c) they measured perceived risk variables regarding gestational alcohol consumption and/or the level of knowledge about the possible teratogenic effects of alcohol. The exclusion criteria were: (a) articles written in a language other than Spanish or English, (b) systematic reviews, conference papers and chapters, (c) target population other than pregnant women or women of reproductive age, (d) articles that did not measure perceived risk variables and/or knowledge of possible effects, and (e) articles focusing on psychoactive substances other than alcohol.

### 2.3. Data Extraction

The authors performed the data extraction. The following information was collected from each article: authors, year of publication, population characteristics (age, educational level and country), questionnaires used and main results.

To analyse the methodological quality of the selected studies, the Critical Appraisal Tools for Qualitative Studies guidelines of the Centre for Evidence-Based Medicine (CEBM) in Oxford were applied [27]. This tool evaluates eight criteria, assigning each a ‘pass’, ‘fail’ or ‘unclear’ rating when the criterion is partially met: 1. Was the qualitative approach appropriate?, 2. Was the sampling strategy appropriate for the approach?, 3. What data collection methods were used?, 4. How were the data analysed and verified?, 5. Was the researcher’s position described?, 6. Do the results make sense?, 7. Are the conclusions drawn from the results justified?, 8. Are the findings transferable to other clinical settings?

The articles were evaluated independently and then discussed jointly to resolve any discrepancies. Finally, each study was given an overall score of 0–8 points, categorising the quality as follows: low quality: 1–3 points, moderate quality: 4–6 points, and high quality: 7–8 points.

## 3. Results

### 3.1. Search and Study Selection

**Figure 1:**
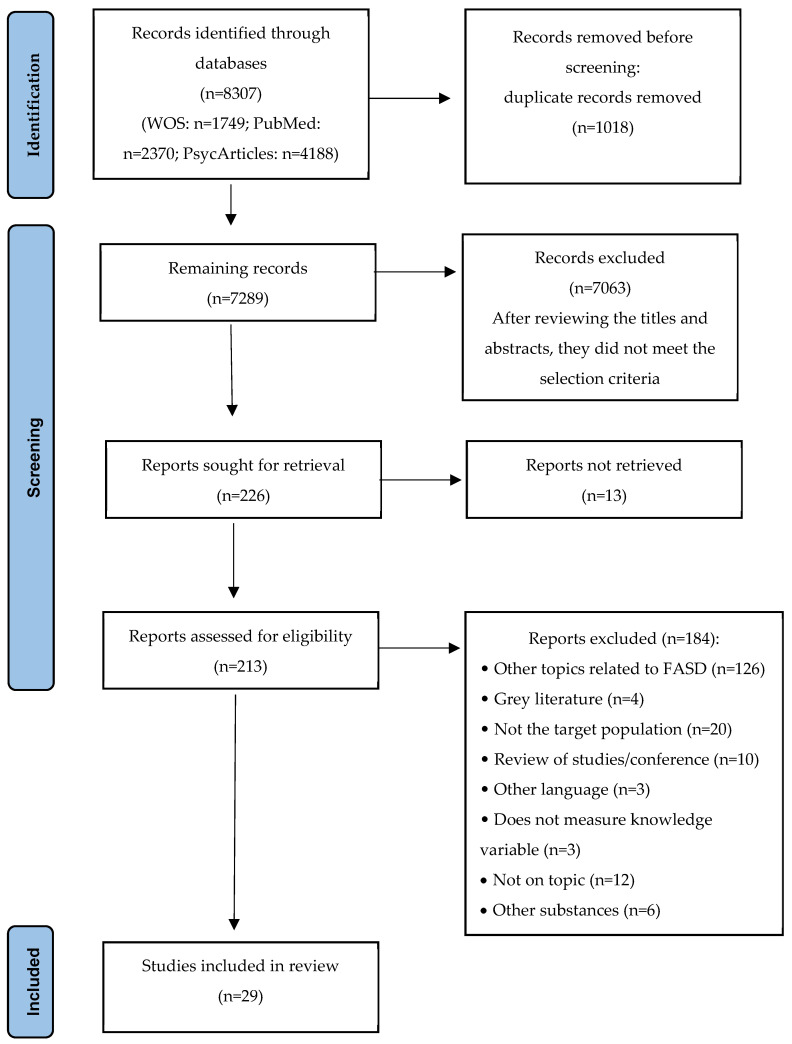
PRISMA flowchart.

A total of 8307 articles were identified, 1018 of which were eliminated due to duplication. After reviewing titles and abstracts, 7063 articles were excluded for not meeting the selection criteria, leaving 226 for full evaluation. Of these, 13 could not be retrieved, so 213 full-text articles were ultimately evaluated. After applying the inclusion and exclusion criteria, 184 articles were eliminated (see Figure 1). Finally, 29 articles were included in the systematic review.

### 3.2. Methodological Quality

According to the Critical Appraisal of Qualitative Studies guidelines [27], 12 articles were classified as high quality (3 with 8 points and 9 with 7 points), while 17 articles were considered of moderate quality (11 with 6 points, 5 with 5 points and 1 with 4 points; see Table 1). No articles were rated as low quality and therefore none were eliminated from the final selection.

### 3.3. Study Characteristics

A total of 17,473 individuals participated in the analysed studies, including 9570 pregnant women, 5018 women with children (4553 of whom were in the postpartum period), and 1222 women of childbearing age (see Table 2). Three studies included a total of 1143 pregnant women, women with children and/or women of childbearing age, but did not specify the exact number in each group [36,41,44].

The women interviewed were aged between 18 and 49 years, except in the studies by Cornelius et al. (1997) and Kristjanson et al. (2007), where the average age was 16.3 years and the lower age limit was 16 years [32,41]. Two studies did not report information on the age of the participants [38,46].

In terms of educational level, the majority of women had received secondary or higher education. However, the study by Balachova et al. (2016) also included participants with primary education, and the study by Kaskutas (2000) included women with no formal education [29,40]. In other studies, the educational level of the participants was not specified [35,38,41,46,50,51,53,55].

According to studies including the variable ‘Gestational Alcohol Consumption’ (GAC, n = 17), the prevalence of alcohol intake during pregnancy ranges from 12.7% to 75%, with consumption being more frequent in the first trimester [28,32,42]. Three of the reviewed studies found that virtually all participants reported alcohol consumption during pregnancy; however, it should be noted that these were convenience samples [30,53,55]. Regarding the frequency of consumption (n = 3), this varied from less than once a month to two to four times a month. In terms of the amount consumed (n = 9), this ranged from less than one drink to more than six per occasion/day. Beer and wine were the most commonly consumed beverages (n = 3). Three studies observed a range of 3–23 drinks per week [39,44,48], while another observed a range of 4–84 drinks per week; however, it should be noted that this is a geographical area with high GAC [53]. Regarding consumption prior to pregnancy (n = 10), assessed in periods ranging from 30 days to one year before conception, between 25% and 87.4% of women reported drinking alcohol. Furthermore, risky consumption (1–6%) has been identified in some cases [41,50,56].

In terms of methodology, most of the studies were cross-sectional in design, with the exception of the longitudinal study by Cornelius et al. (1997) [32]. All studies used ad hoc questionnaires and interviews to assess core variables, such as sociodemographic characteristics (e.g., age, educational level, place of residence and socioeconomic status), obstetric characteristics (e.g., pregnancy planning), GAC (e.g., self-reported frequency of alcohol consumption during pregnancy; Alcohol Use Disorder Identification Test—AUDIT [57]; Alcohol Use Disorder Identification Test-Consumption—AUDIT-C [58]; Tolerance, Worry, Eye-opener, Amnesia, Kut down—TWEAK [59]; Tolerance, Annoyed, Cut down, Eye-opener—T-ACE [60]; Student Alcohol Questionnaire—SAQ [61]); perceived risk (e.g., “Do you believe that it is okay to drink a little during pregnancy?”; “Pregnant women should not drink alcohol”), and level of knowledge about GAC and its effects, including FASDs (e.g., “Indicate whether or not prenatal consumption increases the chance of any of the following: miscarriage, infantile withdrawal symptoms, lower intelligence quotient (IQ)/intellectual disability, …”; “How long FAS lasts”; etc.). Some studies have also evaluated other variables of interest for their research, such as depressive symptoms (Edinburgh Postnatal Depression Scale—EPDS [62], Patient Health Questionnaire—PHQ-9 [63] and PHQ-2 [64]), anxiety symptoms (Generalized Anxiety Disorder scale—GAD 2 [65]), consequences of alcohol intake (Drinker’s Inventory of Consequences—DrInc [66]), social support (Oslo 3-items Social Support Scale—OSSS-3 [67]), level of acculturation (Acculturation Scale for Mexican-Americans—ARSMA-II [68]), and social desirability (Marlowe–Crowne Social Desirability Scale [69]).

Finally, the geographical distribution of the studies shows that most come from Europe (n = 12)—although from very diverse locations—followed by America (n = 6), Asia (n = 5) and Africa (n = 6).

**Table 2:** Sociodemographic, methodological characteristics and main results of the reviewed articles, by geographical continent.

Authors & Sample Country	Participants	Variables/Instruments	Main Results
EUROPE			
McKnight & Merret (1987) Northern Ireland [46]	n = 380 pregnant women.	V/I. Interview at 2 time points: before and after birth: OH consumption, knowledge of fetal OH effects, healthcare professionals who provided information about GAC, intentions regarding future drinking patterns, prenatal classes and health awareness.	Risk perception: majority believed OH harms the fetus. In prenatal interview, 5.8% believed that OH did not affect the fetus, and 10.5% didn’t know. In postnatal interview, the figures were 4.5% and 10%, respectively. Participants were unaware of a safe level of intake. Knowledge: more effects were mentioned in the postnatal interview, but few referred specifically to FAS. Lack of knowledge about specific effects decreased in the postnatal stage. They associated low birth weight more with smoking than OH.
Lelong et al. (1995) France [44]	n = 176 pregnant or postpartum, ≥30 years, ≤16 years of education.	V/I. Sociodemographics; prenatal care; pregnancy course; OH, tobacco and coffee consumption before and during pregnancy; paternal OH and tobacco use; general attitude toward OH; reasons for changing behavior regarding OH and tobacco during pregnancy; and influence of healthcare professionals, family and friends.	Risk perception: 11% believe that drinking ≥3 glasses of wine/day is reasonable, and 17% believe the same for beer. Consuming larger amounts of beer than wine was seen as more acceptable; the acceptable OH limit increased with level of consumption. Little advice to stop drinking was received (7% from professionals, 14% from family). Knowledge: most recognized OH effects on the baby, but sustained heavy drinkers were significantly less likely to mention it
Kristjanson et al. (2007) Russia [41]	n = 899 pregnant and women of childbearing age, 18–43 years.	V/I. 2 questionnaires (based on interview location). Quantity and frequency of OH; OH-related problems and dependence symptoms; demographics, social roles, health, smoking, prescription/illicit drug use; attitudes toward OH intake, expected effects, drinking contexts and companions; contraception, nutrition and diet; and knowledge of the effects of OH on health and pregnancy; pregnancy history; OH consumption before and during pregnancy.	Groups: 3 groups based on interview location: employment centers (n = 308, 31.8 years, SD = 7.2), educational institutions (n = 391, 18.5 years, SD = 3.1), and OB/GYN clinics (n = 200, 26.7 years, SD = 5.6). GAC: women at OB/GYN reported lower current OH consumption (34%) vs. those at educational institutions (86.7%) and employment centers (90.3%). OB/GYN participants drank less and did not exceed 2 drinks/day. Last month: wine was the most consumed beverage (pregnant and non-pregnant women); beer consumption was higher in educational institutions, and liquor in employment centers. Risk perception: over 80% of pregnant women (both abstainers and drinkers) believed that heavy OH use increases risk of miscarriage, intellectual disability, low birth weight, and birth defects. 99.5% of pregnant and 96.7% of non-pregnant women recognized at least 1 harmful effect. Risk perception was higher among abstinent pregnant women vs. drinkers. Knowledge: <50% pregnant women had heard of FAS, but over 70% correctly identified its definition. Knowledge levels were similar between pregnant drinkers and abstainers.
Balachova et al. (2016) Russia [29]	n = 648 women (301 pregnant), 28.2 years (SD = 6.2), primary (36%) and higher education (41%).	V/I. Harmfulness or appropriateness of drinking during pregnancy, knowledge about FASDs, whether they have heard of FASDs, OH consumption (amount, frequency and BD) and risky consumption: T-ACE [60] and TWEAK [59], sexual relations and use of contraceptive methods.	Risk perception: GAC is acceptable (or uncertain) for 40%. 28% believe that there is a safe trimester for consumption, but there is no consensus on which one. 37% think OH type matters: vodka (93%) is the most harmful, wine (73%) is the least harmful. Differences by geographical area: St. Petersburg more permissive. No differences in pregnancy status. Knowledge: only 8% have reliable knowledge about FASDs. 34% have heard of FASDs, of these, 46% associate FASDs with birth defects, 42% with lifelong effects. Less knowledge among pregnant drinkers than non-pregnant. Pregnant women’s knowledge about OH effects and FASDs is related to attitudes and GAC, but this is not associated with risky consumption in non-pregnant women.
Howlett et al. (2017) England [38]	n = 212: 171 pregnant and 41 partners.	V/I. Belief about safe levels of GAC, timing of OH cessation during pregnancy and gestational week of cessation, willingness to undergo blood testing in future pregnancies and meconium testing to detect GAC.	Risk perception: 89.2% believe that OH should not be consumed during pregnancy. 9.4% consider it safe to drink 1–2 units/week, 1.4% believe it is safe to drink +3 units/occasion. GAC: 64.3%. Among those who believed it was not safe, 54.9% continued drinking until pregnancy was confirmed.
Dumas et al. (2018) France [34]	n = 3.603 pregnant and postpartum, 66.5% 25–34 years, 51.9% higher education.	V/I. Sociodemographics, perceived risks associated with OH consumption, knowledge of risks associated with GAC, differences in harmful effects depending on type of alcoholic beverage, awareness and labeling of OH drinks, information on smoking and drinking, internet search for information on OH and tobacco use during pregnancy, information provided by healthcare professionals.	Risk perception: 92.1% perceive 1–2 drinks/day during pregnancy as harmful (higher among drinkers and those with higher education). 21.1% of drinkers and 14.9% of abstainers do not perceive harm in occasional intake. Higher education is associated with perceiving harm from occasional consumption. 89.5% believe that a single BD episode could affect the fetus. 40.8% believe that spirits are more harmful than wine or beer (more common among those with lower education). 65% reported reading about OH and tobacco in pregnancy health booklet. Among women who drank before pregnancy, 30.2% received a recommendation to abstain or reduce consumption, compared to 63.2% of smokers. Knowledge: 56.4% identified only 1 effect: brain damage (34.2%), malformations (30.2%), growth delay/low birth weight (28.6%). Lack of awareness was greater among those aged ≥35, with lower education levels and among single women.
Corrales-Gutierrez et al. (2019) Spain [33]	n = 426 women, 31.9 years (SD = 5.3), 45.5% secondary education.	V/I. Sociodemographic and obstetric factors; general belief about whether OH consumption harms the mother or fetus; type of risk from OH; belief about the duration of consequences; belief about the risk of OH consumption according to quantity, frequency and type of drink; and frequency of OH consumption (AUDIT [57]).	GAC: No: 75.4%; Yes: 14.6% (once or less/month), 8.4% (2–4 times/month), 1.2% (2–3 times/week). Risk perception: 69.7% believe that any consumption is harmful; 0.4% believe it is harmless; 3.9% believe that a small daily amount is harmless; 6.6% see no risk in moderate consumption; 48% believe there is a risk to the baby; 42.6% believe there is a risk to both baby and mother. Types of beverages in GAC: perception of harm from beer (31.5%), wine (38.4%), spirits (88%); perception of small daily amount as not harmful: beer 14.8%, wine 5.9%, spirits 1.9%. Knowledge: 27.1% unable to specify any; malformations (72.9%), developmental problems (24.6%), premature birth (2.5%). Duration of risk: 48.1% always, 15.5% brief, 27.5% do not know.
Franco et al. (2020) Spain, France & Portugal [36]	n = 68: Spanish women (n = 30, 34.26 years, SD = 3.67), French (n = 20, 33.3 years, SD = 4.6), and Portuguese (n = 18, 30.67 years, SD = 5.21), all pregnant/mothers, majority higher education.	V/I. AUDIT [57]: prior to and during pregnancy, sociodemographics, OH consumption, information and knowledge about GAC consequences, and assessment and selective prevention (information available in primary care context), indicated and universal prevention.	GAC: in Spain, 66% reduced consumption and 34% abstained completely, in France and Portugal total abstinence ranged between 70–75%. Risk perception: women who drink believed that low and occasional doses do not harm the fetus. Social environment supported GAC on festive occasions. Knowledge: was limited and often incorrect (e.g., believing abstinence is only necessary during first trimester).
Oechsle et al. (2020) Germany [47]	n = 209 pregnant, 31.7 years (SD = 4.6), 54% higher education.	V/I. Sociodemographics; knowledge and attitude toward OH consumption; smoking, nutrition and supplementation; physical activity, oral health and medication.	Risk perception: 10.8% believe that only regular intake causes FASDs, 77.9% think that even small amounts affect the fetus, 3.6% believe that occasional champagne consumption is harmless. Knowledge: 97.1% are aware of the recommendation to avoid OH during pregnancy, but 31.8% are unaware of specific adverse health effects. 87.7% believe that any OH exposure can cause lifelong problems.
Ujhelyi et al. (2022) England [52]	n = 20 women: 6 pregnant and 8 mothers (≥30 years, higher education), and 7 healthcare professionals (>18 years).	V/I. GAC and OH consumption during motherhood; perceived risks, benefits and short- and long-term effects of OH; reasons for and against OH consumption during pregnancy and motherhood; questions for professionals about OH use in mothers and pregnant; sociodemographics and questions for obstetricians, AUDIT [57], PHQ-2 [64], and GAD-2 [65], birth experience and trauma, and other life traumatic events.	GAC: 1 pregnant and 87.5% of mothers. AUDIT scores: pregnant = 0; mothers < 7. Most stopped drinking upon pregnancy confirmation, some consumed low levels (on special occasions or 1–2 times). Risk perception: most pregnant and mothers believe that the safest option is to avoid or limit OH, while others think low levels are acceptable due to a perceived lack of convincing evidence about the risks. All professionals recommend abstaining from OH. Knowledge: OH use during pregnancy is associated with risks to the baby (birth defects, FASDs, developmental problems), to the mother, and to family relationships.
Piotrkowiz et al. (2023) Poland [49]	n = 471 women (54.6% had been pregnant), 15–49 years, 72.4% higher education.	V/I. Sociodemographics, knowledge of GAC and FASD risks, personal experience with OH and its prevention during pregnancy, religion, reliable sources of information on GAC, and whether they were encouraged to drink during pregnancy and by whom.	Groups: Generation X (ages 42–49, 9.6%), Generation Y (ages 24–41, 55.6%), and Generation Z (ages 15–25, 34.8%). Risk perception: nearly 100% of Generations Y and Z recognized the teratogenicity of OH, while 4% of the older generation believed it was not very harmful. Younger generations received more OH warnings. 12% of older women were advised to drink occasionally by professionals, but none of the younger women were. Knowledge: Generations Y and Z gave more correct responses, but younger individuals (Z) showed less overall knowledge. 97% of Generation Y, 89% of Generation X, and 84% of Generation Z had heard of FASDs. Fewer than 50% answered correctly about the diagnosis of FAS. Belief that only large amounts cause FAS: X (24%), Y (19%), Z (22%). Generation Z showed lower awareness that abstaining from OH is a form of prevention. Knowledge was higher in urban areas. No significant relationship between perception of fetal OH risk and smoking, religion, or general OH consumption frequency.
Zahumensky et al. (2024) Slovakia [56]	n = 402 postpartum, 32.4 years, 78.6% higher education.	V/I. Demographics, awareness of the effects of GAC, GAC by trimester, pregnancy planning [70].	GAC: 73.1% did not drink, while 26.9% consumed during at least one trimester. None of the women who were abstinent prior to pregnancy consumed during gestation. Around 60.5% of those who drank before pregnancy stopped drinking during pregnancy. Knowledge: 74.6% knows effects of OH during pregnancy.
THE AMERICAS			
Testa & Riefman (1996) USA [51]	n = 159 pregnant, 27.3 years (SD = 5.68).	V/I. GAC, previous pregnancy outcomes, lifetime OH-related problems, perceived risk of GAC, socioeconomic status (Four Factor Index of Social Position [71]), social desirability (Marlowe–Crowne Social Desirability Scale [69]).	Risk perception: first-time mothers reported higher perceived risk and lower OH consumption. Having had a previous healthy pregnancy was associated with lower risk perception and increased OH intake during the current pregnancy.
Cornelius et al. (1997) Pennsylvania [32]	n = 415 pregnant, 16.3 years, primary education (56.7%).	V/I. Two-stage interview: (1) Year prior to pregnancy and first trimester: OH and other drug use, demographics, psychosocial and medical factors, sexual history, conception-recognition-confirmation. (2) 24–36 h postpartum: OH/drug use in second and third trimesters, nutritional history, mental health, maternal perception of herself and the newborn, drinking consequences, knowledge/attitudes about OH, paternal initiation and use, knowledge of effects of use, scale of attitudes towards OH.	GAC: 47% (1st trimester), 12% (2nd trimester) and 8% (3rd trimester), <1 drink/day. Knowledge: greater knowledge about the effects of OH on the fetus was associated with lower consumption and greater reduction between trimesters, 99% with high knowledge reduced/stopped drinking (1st–3rd trimester) vs. 90% with low knowledge.
Kaskutas (2000) USA [40]	n = 321 pregnant, <25 years. No formal education.	V/I. Responses to 5 sources of health warnings about GAC (e.g., point-of-sale signage: “was the message understood?”), perception of GAC risks (including congenital defects associated with FAS), OH consumption (12 months prior to pregnancy recognition and after recognition).	GAC: 27% drank OH after pregnancy recognition (30% African American, 21% Native American), quantity: ≥1 standard drink/day. 18% daily consumption, 39% drank + 3 drinks/day, 14% +5 drinks/day (higher among African American). Risk perception: African American women showed lower accuracy, and those who drank beer believed it was safer than other alcoholic beverages. Heavy drinkers were less aware of the need to reduce consumption, and wine drinkers believed it was safer. <20% believed it was “too late” to reduce OH, wine was perceived as safer. 83% had seen ads about GAC, 43% had seen point-of-sale signs, and 96% had had conversations on the topic. Knowledge: 25% identified at least 1 birth defect, and only 20% recognized OH as the cause.
Chambers et al. (2005) Canada [31]	n = 100 pregnant,18–40 years (SD= 6.2), 38% higher education.	V/I. OH consumption 3 months prior to pregnancy confirmation and after confirmation; amount, frequency and type of OH; TWEAK [59]; sociodemographic characteristics; acculturation level (ARSMA-II [68]); health history and knowledge of the effects of OH on the fetus and of warning messages.	Risk perception: drinkers, including BD, saw warnings on packaging and in public places. Knowledge: 55% had heard of FAS, and 38% described it correctly. Female drinkers were aware of FAS and its risks vs. non-drinkers.
Zabotka et al. (2017) USA [55]	n = 11 biological mothers (43 years) of children with full FAS (17 years).	V/I. Substance use and feelings, thoughts, and reactions to the etiology of the disorder.	Knowledge: lack of control (alcoholism–disease model) and lack of knowledge about the risks of OH during pregnancy; advice from friends, family or professionals to drink (“nothing happens if you drink during the first weeks”); or unawareness of the pregnancy.
Caires & Santos (2020) Brazil [30]	n = 17 GAC women, <30 years, secondary education (58.8%).	V/I. Sociodemographics, OH treatment time, abstinence, reproductive history (pregnancies, abortions, nº of children and type of delivery), GAC and prenatal care received.	Knowledge: lack of information about the harm that OH can cause to her and her child, some notion that OH can harm the fetus. Fetal malformation is only related to a physical defect, and the possibility of neurocognitive disorder is not considered.
ASIA			
Lee et al. (2010) Republic of Korea [43]	n = 646 pregnant, 31.56 years (SD = 3.91), years of education = 15 (SD = 2.17).	V/I. Demographics (age, education level, gestational weeks, pregnancy or miscarriage history, occupation, annual household income, pregnancy planning and smoking habits); OH consumption: AUDIT-C [58], frequency and quantity; knowledge of GAC and FAS effects.	GAC: 83.6% did not drink. Among those who did: 12.4% less than once/month, 3.6% drank 2–4 times/month, and 0.3% 2–3 times/week. 12.7% consumed 1–2 drinks, 1.2% 3–4 drinks, 0.9% 7–9 drinks, and 0.6% 10 drinks/occasion. Beer was the most commonly consumed beverage during pregnancy. 79.88% were classified as abstinent and 20.12% as drinkers during pregnancy. GAC profile: lower education level, lower pregnancy planning, and higher pre-pregnancy OH use. Knowledge: ~50% had heard of FAS, drinkers were less informed about OH impact.
Kim & Park (2011) Republic of Korea [42]	n = 221 postpartum, 32.97 years (SD = 3.67), secondary education (76%).	V/I. Quantity and frequency of OH (6 months prior to pregnancy and in each trimester), SAQ [61], FAS knowledge.	GAC: 12.7% drank during pregnancy, mostly in first trimester (7.7%) and with their partner (60.7%). Risk perception: common misconception was that GAC is acceptable in certain quantities. No differences found based on information source. Only 2.3% received information from nurses about OH, and 1.8% about FAS. Knowledge: mean OH knowledge score: 15.56/29, mean FASD knowledge score: 3.63/8. GAC was not related to knowledge of OH or FASDs.
Senecky et al. (2011) Israel [50]	n = 3.815 recent mothers, 30.3 years (SD = 5.3).	V/I. Sociodemographics, GAC education received from professionals, quality and quantity of the information, other sources, GAC knowledge, attitudes toward women who drink during pregnancy, amount of OH consumed before conception and in the last 3 months of pregnancy, BD and T-ACE [60].	GAC: 17.1% (Jewish and Christian), 23% knew pregnant women who drank. 1.4% engaged in BD in the 3 months prior to pregnancy (0.8% in 3rd trimester). Risk consumption: 0.68%. Risk perception: Among non-drinkers, 25% received education about OH and 52% about nutrition; among drinkers, 17% and 37%, respectively. Knowledge: 71.6% stated that one should not drink during pregnancy, 21.4% believed it is safe to consume up to 2 drinks/week.
Hen-Herbst et al. (2021) Israel [37]	n = 802 pregnant, 30.76 years (SD = 4.58), 53.2% higher education.	V/I. Sociodemographic and obstetric variables, OH intake before and during pregnancy, information sources on GAC, FASD knowledge and its effects, other habits (nutrition, physical activity, tobacco).	GAC: 67.2% drank in 2 months before pregnancy recognition. 12% consumed OH during pregnancy, and among these: 63.8% drank up to half a glass/occasion, 33% 1 glass, 2.1% 2 glasses, and 1.1% +2 glasses. 86.6% reduced consumption after pregnancy recognition. 28.1% knew pregnant women who drank. Risk perception 39.5% had not received information about GAC. Those who had: doctors (37.4%), nurses (17.2%), social media (5.9%). Knowledge: lower among women who drank in the 2 months before pregnancy, those with more children and those with secondary education.
Huang et al. (2023) Philippines [39]	n = 100 postpartum, 28.6 years (SD = 5.3), 61% secondary education.	V/I. Perceived importance of maternal health during pregnancy, knowledge that tuba is an alcoholic beverage, self-reported behaviors regarding OH consumption before and during pregnancy, perceived risks/benefits of GAC, OH-related behaviors in infants, perceived risks/benefits of OH consumption in infants, expectations of behavior change if informed about OH risks, influence of external sources (family, friends, and medical personnel) on tuba intake, and perceived interest in obtaining additional information on maternal and fetal health.	GAC: 75% drank tuba or other alcoholic beverages during pregnancy (all consumed tuba, and 5.3% also drank beer). 36% drank between 3–5 glasses/week. Risk perception: nearly 100% considered nutrition, physical exercise, medical visits and reducing/quitting tobacco during pregnancy to be important/very important vs. 60% who considered reducing GAC to be important/very important. Around 50% had not been informed by a doctor about fetal effects of tuba, and 31.1% did not see a doctor during pregnancy. 98% stated that if they had been informed about the effects of fetal OH, they would have stopped drinking. 100% expressed a desire to learn how to have a healthy pregnancy and keep their baby healthy. 59% reported that their families or friends encouraged them to drink tuba during pregnancy. Knowledge: 75% believed that tuba does not contain OH, and 48% believed that tuba or other alcoholic beverages are good for the fetus and mother. 15% gave tuba to their baby during the first year of life (1 tablespoon/day).
AFRICA			
Eaton et al. (2014) South Africa [35]	n = 1.047: 565 men (20% had a pregnant partner), 28 years (SD = 8.96); and 482 women (14% pregnant), 30.4 years (SD = 11.63).	V/I. Demographics and pregnancy status, OH consumption (frequency, quantity, HED, and current consumption in public venues), beliefs about FASDs.	GAC: high-risk patterns were similar between pregnant and non-pregnant women. Pregnant: 53.8% drinking 2–4 times/month, 56.8% consumed 3–4 drinks/occasion, 59.6% reported HED (>6 drinks/occasion). Risk perception: 40% of pregnant and 29% of non-pregnant people disagreed with avoiding GAC. 37% of pregnant and 21% of non-pregnant did not agree that OH use causes fetal harm. 50% of pregnant and partners believed that OH can be consumed without harming the fetus. 31% of pregnant considered ≥3 drinks/day to be safe (vs. 8% of non-pregnant). 35% of pregnant women believed that drinking monthly does not harm the fetus. Lower risk perception was associated with higher drinking frequency.
Onwuka et al. (2016) Nigeria [48]	n = 380 pregnant, 71.3% 26–35 years, 72.1% higher education.	V/I. Sociodemographics; GAC; awareness of the OH effects on babies (including FASDs); initial source of information about awareness, type and amount of OH consumed; reasons for drinking; and willingness to quit after counseling.	GAC: 22.6% drank during pregnancy; of these, 82.6% drank occasionally (53.5% classified as BD), and 17.4% drank regularly. Average consumption: 5.3 ± 2.7 units/week (68.9 ± 35.23 g ethanol). Beverages: strong beer (62.8%), lager beer (55.8%), red wine (37.2%), palm wine (32.6%), refined gin/liquor (9.3%), and local gin (4.7%). Risk perception: after counseling about GAC effects, 86% said they would be willing to stop drinking, while 13.9% would continue due to habit. Knowledge: 35.5% were aware of the fetal risks of OH, 64.5% were unaware.
Watt et al. (2016) South Africa [53]	n = 24: pregnant (n = 9) or postpartum (n = 15), 27 years.	V/I. Experiences of gestational OH consumption, norms and attitudes toward maternal OH use, FASD knowledge.	GAC: nearly all participants reported risky patterns of GAC (including BD). Among those who reported risky patterns, they described their consumption as increasing (n = 3), decreasing (n = 3), or unchanged (n = 11) during pregnancy. Risk perception: more than half had received messages advising against drinking during pregnancy. Some believed that only certain types of OH (e.g., spirits) affect the fetus, they placed greater trust in their own “intuition” and optimism about the effects than in external (clinical or family) information. Knowledge: few identified specific effects, most were unfamiliar with the term and had limited or inaccurate information about fetal OH effects.
Louw et al. (2018) South Africa [45]	n = 129 women, 29 years (SD = 6.45), years of education = 9.4 (SD = 2.62).	V/I. Risk perception regarding OH use during pregnancy (2 measures: one related to personal risk and another to perceived general community risk), demographics, pregnancy history and adverse birth outcomes, smoking history, FASD knowledge, AUDIT [57].	Risk perception: personal risk perception averaged 4.29/5 and showed a positive correlation with general risk perception (59.5% of variance explained). Predictors of personal risk perception: perceived ease of quitting OH, FASD knowledge, general risk perception, and perceived easy for others to quit drinking. Personal risk perception emerged as the strongest predictor in multivariate models. Predictors of general risk perception (excluding personal risk): FASD knowledge and perceived easy of quitting alcohol (24% of the variance explained). Knowledge: mean score of 8.75/10.
Addila et al. (2021) Ethiopia [28]	n = 1216 pregnant, 27.2 years, secondary education or above (61.76%).	V/I. Demographics, AUDIT-C [58], EPDS [62], OSSS-3 [67], knowledge and attitude about GAC, socioeconomic status, and OH consumption.	GAC: 30.26%: most in trimester 1 (77.2%) and between 1–2 drinks (71.2%), mainly fermented. 13.32% have depression and of these, 37.04% consumed gestational OH. Risk perception: only 15.26% received prenatal information about the effects of GAC. Knowledge: 43.91% were unaware of the risks of GAC. 73.27% had low knowledge of the effects of GAC.
West et al. (2024) Tanzania [54]	n= 541 pregnant and non-pregnant, 40.9 years (SD = 16), 36.6% higher education.	V/I. Sociodemographics, OH consumption, AUDIT [57], DrInc [66], and PHQ-9 [63].	GAC: 56.9% of pregnant did not consume OH, and among the 42.2% who did, 32.8% drank 1–2 drinks and 9.5% consumed 3–4 drinks. Non-pregnant tended to consume 3–4 drinks. AUDIT scores were similar between 2 groups (0–2 in pregnant, 0–4 in non-pregnant). Younger non-pregnant people reported more OH-related consequences. Risk perception: 70% believed that 0 drinks are the safe amount during pregnancy.

ARMSA-II: Acculturation Rating Scale for Mexican Americans-II; AUDIT: Alcohol Use Disorders Identification Test; AUDIT-C: Alcohol Use Disorders Identification Test–Consumption; BD: binge drinking; DrInc: Drinker’s Inventory of Consequences; EPDS: Edinburgh Postnatal Depression Scale; FAS: fetal alcohol syndrome; FASDs: fetal alcohol spectrum disorders; GAC: gestational alcohol consumption; GAD-2: Generalized Anxiety Disorder-2; HED: heavy episodic drinking; OB/GYN: obstetrics and gynecology; OH: alcohol; OSSS-3: Oslo 3-item Social Support Scale; PHQ-2: Patient Health Questionnaire-2; PHQ-9: Patient Health Questionnaire-9; SAQ: Student Alcohol Questionnaire; SD: standard deviation; T-ACE: Tolerance–Annoyed–Cut down–Eye-opener questionnaire; TWEAK: Tolerance–Worry–Eye-opener–Amnesia–Kut down questionnaire; V/I: Variables/Instruments.

### 3.4. Alcohol Consumption During Pregnancy: Perceived Risk and Knowledge of Possible Effects Perceived Risk of Gestational Alcohol Consumption

Most women believe that alcohol can harm the fetus, although the level of risk attributed varies depending on the amount, frequency and timing of pregnancy. While 12–40% believe some alcohol consumption is acceptable during pregnancy, nearly one-third (30%) consider any intake harmless, and around one in five underestimate the risks of low or occasional drinking [29,33,34,47,54]. A substantial minority also perceive certain trimesters or small daily amounts as safe, and up to 9% of pregnant women accept one to two units per week, with a smaller proportion tolerating occasional higher-intensity consumption, including champagne [29,33,38,47,50]. These findings reveal the persistence of permissive beliefs and misperceptions.

Female drinkers tend to minimise the risk associated with low quantities or occasional consumption: 21.2% of drinkers and 14.9% of abstainers consider this pattern to be harmless [34,36]. Similarly, 11–20% perceive that only regular or large quantities cause FAS [47,49]. Longitudinal data from McKnight and Merret (1987) indicate a modest reduction in risk denial over time: in prenatal interviews, 5.8% stated that alcohol does not affect the baby and 10.5% were uncertain, compared with 4.5% and 10%, respectively, in postnatal follow-ups [46]. Consistent with this, over 80% of pregnant women recognise that excessive alcohol consumption increases the risk of miscarriage, neurodevelopmental impairment, low birth weight and malformations [41]. These perceptions contrast sharply with beliefs regarding moderate or non-excessive intake, which remain permissive.

Some studies reveal particularly permissive beliefs. For example, in a study by Eaton et al. (2014) in Cape Town, where a large proportion of pregnant women drink alcohol, 40% of pregnant women and 29% of non-pregnant women disagreed with the idea of avoiding alcohol during pregnancy [35]. Furthermore, 37% and 21% of pregnant and non-pregnant women, respectively, did not believe that fetal harm existed. Up to 31% of pregnant women considered it safe to consume three or more drinks per day, and 35% believed that drinking once a month posed no risk. Perceptions also varied by beverage type: 37–41% considered distilled spirits more harmful than fermented beverages, with 88% identifying spirits as harmful, 38% perceiving wine as risky, and 32% perceiving beer as harmful [29,33,34]. However, 15% believe that the daily consumption of small amounts of beer poses no risk, compared to 6% for wine and 2% for spirits [33]. Regarding quantity, 17% considered three or more glasses of beer per day acceptable, and 11% felt similarly about wine, whereas only 6% reported that no alcoholic beverage is safe during pregnancy [33,44]. In the Philippines, misconceptions about locally consumed beverages are notable. In a study on tuba, a traditional palm wine, 75% of women believed it contained no alcohol, and 48% thought it had beneficial effects for both mother and fetus [39]. These findings underscore the persistence of permissive beliefs, the influence of beverage type on perceived risk, and the potential for cultural and local drinking practices to exacerbate prenatal alcohol exposure.

Studies that took women’s attitudes towards alcohol intake during pregnancy into account, found that 54% of participants had a negative attitude towards it [28]. A more intolerant attitude towards alcohol was associated with a lower frequency of consumption, a lower prevalence of BD episodes, and a reduction in the negative consequences of consumption [32]. The impact of attitude on behaviour was evident: 91% of women who opposed consumption stopped drinking or reduced their intake, compared to 84% of those with more permissive attitudes, in both the pre-pregnancy period and the first trimester. This difference was maintained throughout the three trimesters of the follow-up: 100% of women with anti-consumption attitudes reduced their intake, compared to 92% of those with pro-consumption attitudes [32].

### 3.5. Knowledge of Possible Efects of Gestational Alcohol Consumption

Knowledge of the risks associated with GAC remains limited. Across studies, between 44% and 73% of women reported little or no awareness of these risks [28,47]. While approximately 48% perceive GAC as harmful to the baby, only 43% identify risks to both mother and child [33,52]. Awareness of FASDs is similarly low: although 34–55% of participants had heard of the syndrome, only 8–38% were able to describe it accurately [29,31,41,43]. High levels of general knowledge about FASDs are context-dependent, as illustrated by an average score of 8.75 out of 10 in a study conducted in Cape Town [45].

Despite some awareness, misconceptions persist. In certain communities, prenatal alcohol use is normalized because no observable adverse effects in newborns have been reported [54]. Although some risks were acknowledged, such as premature birth, blindness and neurological and cognitive impairments, there was also a belief that alcohol intake during pregnancy could have benefits for the fetus and mother, such as ensuring a strong baby or reducing the pain of childbirth.

Despite this partial knowledge, between 84% and 97% of women are aware of alcohol’s teratogenic potential [47,49]. However, only a minority (approximately 20–25%) can identify any specific defect associated with FAS or recognise maternal alcohol consumption as its direct cause [40]. Beliefs regarding the permanence of effects vary: 48–87% consider them permanent, 16% view them as temporary, and 28% are unsure [33,47].

Information from healthcare professionals is often insufficient. Between 15% and 75% of participants reported not having been informed about the risks of alcohol consumption during pregnancy [28,37,39,50], even when nutritional advice is provided. While nearly all participants recognize the importance of factors such as nutrition, exercise, medical visits, and smoking cessation, only 60% identify reducing alcohol as important [39]. Misattribution of outcomes persists: some women link low birth weight to smoking rather than alcohol [46].

Knowledge of official abstinence guidelines is limited, and information availability is sparse [36,44,53]. Nevertheless, nearly all women express willingness to learn about healthy pregnancies, with 98% stating they would abstain if informed of fetal alcohol risks [39].

Exposure to warning messages on alcoholic beverage packaging is uncommon, with only two studies reporting high visibility: 99% of participants reported having seen recommendations to abstain [34], and 97% of the sample suggested avoiding alcohol during pregnancy [47].

A range of factors can influence women’s knowledge of the effects of alcohol on pregnancy and FASDs. At least three studies have identified low educational attainment as a predictor of lower knowledge about the risks of FAS [28,33,37]. Likewise, several studies have highlighted widespread lack of knowledge of the effects of gestational alcohol, particularly among women with children, active drinkers and those in later life [37,44,49]. Active or pre-pregnancy alcohol intake has been associated with lower awareness of FASDs or an underestimation of its risks [29,43,44,53]. Additionally, both younger and older age groups have been associated with higher levels of unawareness [49], as well as a lack of health coverage or low income [47].

Studies by Onwuka et al. (2016), Piotrkowicz et al. (2023) and Huang et al. (2023) have shown that exposure to warning messages or professional counseling significantly improves knowledge and the intention to quit drinking [39,48,49]. However, it has also been observed that, in some contexts, knowledge does not necessarily lead to reduced consumption [41,42].

On the other hand, several studies indicate that GAC is influenced by multiple individual, contextual and social factors. At the individual level, one of the most consistent predictors is a history of alcohol consumption prior to pregnancy [37,42,44,48,51,56]. Similarly, young maternal age (under 30 years) is associated with a higher probability of drinking during pregnancy [31,33,37,44,48,49,50]. Low educational level is another common factor [28,33,34,44,48,50]. Concurrent risk factors include smoking and primiparity [31,44,50], a variable associated with higher consumption [37,48,50].

Other relevant factors include limited knowledge of the effects of GAC [56], lack of pregnancy planning [44,56], partner alcohol consumption [28,44,50], social pressure [39], social acceptance of drinking in festive settings [28,36] and the absence of religious practice or membership of certain religious groups [37,50]. It has also been reported that GAC may be influenced by personal motivations such as emotional coping, perceived permissive social norms, individual decision-making, as well as by a positive or neutral perception of alcohol [28,52].

A lower perception of risk associated with GAC is also associated with higher consumption [33,35], and it has also been found that GAC tends to increase when the consequences of the first pregnancy are perceived as positive or when the consequences of gestational consumption are not appreciated [51,54].

At the community level, the most notable factors include higher levels of acculturation and income [31], unemployment and school dropout [44], delayed initiation of prenatal care [44], and limited exposure to preventive messages regarding GAC [28].

## 4. Discussion

This systematic review provides a comprehensive overview of two key aspects related to alcohol consumption during pregnancy: risk perception among pregnant women or women of childbearing age, and their knowledge of the potential consequences for offspring.

The results reveal substantial variability in reported prevalence of alcohol use during pregnancy, partly attributable to differences in data collection methods. Studies using biomarkers to determine alcohol intake during pregnancy report higher consumption rates than those relying on self-report [13], suggesting a possible underestimation due to social desirability bias. Regardless of measurement method, gestational alcohol use remains alarmingly common, particularly in the first trimester—a critical period of embryonic development when organogenesis and neurological development are especially vulnerable to teratogenic effects [52].

Pre-pregnancy alcohol consumption emerges as the most consistent predictor of alcohol use during pregnancy [15,16]. The high prevalence of alcohol consumption among women of childbearing age, combined with the frequency of unplanned or unrecognized pregnancies, increases the likelihood of early fetal exposure. These findings underscore the urgent need for preventive and educational strategies targeting all women of reproductive age, particularly those engaging in regular or hazardous drinking.

Although the harmful effects of alcohol during pregnancy are well established, permissive beliefs persist, and uncertainty remains regarding “safe” limits of consumption [72]. While most women acknowledge that alcohol can harm the fetus, perceptions of risk vary according to the amount, frequency, and timing of consumption. Misconceptions persist, with some women considering occasional drinking acceptable, and others perceiving specific gestational stages as “safe” [29,36,39,47,52,73,74,75]. These attitudes are more frequent among women with a history of alcohol use [14,15,16,28,47], who are also more likely to minimize the risks of low-intensity consumption and to believe that only heavy, chronic use is harmful [34,36]. These beliefs persist despite robust evidence that no safe threshold of alcohol intake exists during pregnancy [76].

Cultural and contextual factors further shape perceptions. In European countries like France, Spain and Portugal, the social normalization of alcohol complicates efforts to promote abstinence [36,72,75]. Beverage type also influences perceptions: spirits are widely recognized as harmful, whereas wine and beer are often considered less risky [29,33,34,47]. These cultural framings, where alcohol is viewed as part of lifestyle, hinder the adoption of complete abstinence—even among women previously informed about potential harms. Effective prevention strategies must therefore be context-sensitive and culturally tailored.

It is also important to address the lack of knowledge among some women regarding whether a drink contains alcohol. Misconceptions about local drinks are striking—for instance, Filipino women surveyed did not know that palm wine (tuba) contains alcohol, and considered it beneficial for mother and child [39]. Such misconceptions not only increase maternal exposure but also encourage consumption among offspring, compounding risks.

The persistence of these misconceptions represents a major challenge for clinical practice. Clear and consistent health messaging is essential [77,78,79]: there is no safe level of alcohol consumption during pregnancy, including occasional or low-intensity use. Women’s knowledge about specific consequences of prenatal alcohol exposure remains limited and often inaccurate [28,47]. While many recognise alcohol as teratogenic [47,49], few identify specific disorders such as FASDs, instead associating harms mainly with visible malformations rather than neurodevelopmental deficits [33,34], and believe the effects are temporary or cannot specify their duration. This knowledge gap may reflect insufficient counselling by healthcare professionals, as many women report not receiving professional guidance on the risks of alcohol during pregnancy [37,50]. When counseling is provided, most women indicate willingness to abstain, highlighting a missed opportunity for effective preventive action [39,46].

In summary, a paradox is highlighted: while general awareness that alcohol can harm the fetus has improved, detailed understanding of its full consequences remains limited, misconceptions persist, and cultural and structural barriers continue to normalize consumption. Effective prevention therefore requires an integrated, multilevel strategy that strengthens health professionals’ role, adapts interventions to sociocultural contexts, and communicates a clear evidence-based message: alcohol is unsafe at any level during pregnancy.

One of the most consistent predictors of low knowledge levels is educational attainment [28,33,37], which highlights the importance of tailoring preventative messages to the sociodemographic profile of the population. At the same time, a more pronounced information gap is observed among women who have a history of consumption or who have children [37,44,49], which could be due to a lower perception of vulnerability or the normalisation of alcohol consumption, particularly if no obvious adverse consequences were experienced in previous pregnancies. Studies agree that active or pre-pregnancy alcohol consumption is associated with a higher risk of gestational alcohol intake and an underestimation of its effects, as well as less familiarity with FASDs [29,43,44,53,80]. Furthermore, factors such as a lack of access to health services or a low income can limit opportunities to receive reliable information [47], leading to significant inequalities in maternal and child health.

On a positive note, exposure to warning messages and professional counselling increases knowledge and intention to abstain [48,49], though knowledge alone does not always translate into behaviour change [41,42]. This underscores the need for multidimensional strategies that integrate information with attitudinal, motivational, cultural, and social components.

Individual factors such as pre-pregnancy alcohol use, young maternal age, low educational attainment, smoking, primiparity, and unintended pregnancy are consistently associated with higher risk of gestational drinking [14,15,16,37,42,44,48,51,81]. These variables reflect a profile of greater vulnerability that should be early identified in primary care and obstetric services. It is important to conduct high-quality research to identify the combination of characteristics that best define a potential alcohol consumer during pregnancy. Establishing such a profile would enable not only the tailoring of preventive measures but also their more effective targeting toward the most vulnerable populations.

Personal attitudes play a crucial role: neutral or positive views on gestational drinking, or use of alcohol for coping, increase the likelihood of consumption [28,52]. Here, it is crucial that healthcare professionals are trained to explore ambivalence and reinforce motivation for change. It would also be beneficial to offer guided psychoeducational group interventions combining information, mutual support and healthy coping strategies.

At the relational level, the influence of the immediate environment is also significant. The social acceptance of alcohol consumption in festive contexts, as well as alcohol intake by partners, family or friends, contributes to the normalisation of gestational consumption [28,36,44,50]. Preventive interventions must therefore target not only women but also their social environments.

Finally, structural and community factors, such as limited exposure to preventive messages and late initiation of prenatal care [28,44,82], highlight the need for stronger public health policies and expanded community-based prevention [83,84,85].

In addition to these factors, a temporal analysis of the reviewed articles reveals a progressive shift in risk perception over recent decades. In the 1980s–1990s, knowledge of teratogenic effects was limited, and moderate consumption—particularly of wine and beer—was often normalized in Europe and North America. From the 2000s onward, a trend toward reduced consumption after pregnancy confirmation and increased risk perception emerged, though cultural differences persisted regarding the perceived safety of certain types of beverages. Between 2010 and 2020, campaigns and labeling in Europe and North America contributed to greater recognition that no safe level of alcohol exists, yet detailed knowledge about FASDs remained scarce. Most recently (2020–2024), European studies show high risk perception among younger generations, although with persistent gaps in knowledge about the specific effects of prenatal exposure and the adoption of cultural beliefs that downplay the risk to the fetus of consuming small amounts of alcohol. In addition, in the EU region, despite moderate decreases observed in recent years, the prevalence of alcohol consumption among women remains higher than the global prevalence. This is believed to be due to increased economic wealth, changes in gender roles, lifestyles, and alcohol accessibility [86]. In North America, socioeconomic and ethnic disparities remain, with certain groups still perceiving fermented beverages as safer than distilled spirits. In contrast, in Africa and in certain Asian contexts, high-risk consumption patterns are evident, with elevated prevalence of intake even during pregnancy and a clearly lower risk perception. In these settings, limited access to health information and the influence of cultural beliefs facilitate the persistence of consumption, as seen in the Philippines with traditional beverages. However, several African studies show that risk perception improves following educational interventions, highlighting the preventive potential of culturally adapted awareness programs.

Overall, the temporal evolution reflects a sustained improvement in risk perception and a reduction in consumption across much of Europe and North America, while regional and cultural gaps persist in Africa, Latin America, and Asia. These differences underscore the need for preventive interventions that are sensitive to cultural contexts, as well as for strengthening specific knowledge about FASDs even in regions where abstinence is already predominant.

In summary, achieving a substantive decrease in this behavior requires addressing not only individual, attitudinal and behavioral factors, but also the broader social, cultural, and structural determinants that sustain it. Accordingly, the implementation of multidimensional prevention frameworks—encompassing psychoeducational interventions, psychosocial support, and regulatory policies—appears essential, with careful consideration of the contextual specificities in which such strategies are operationalized.

Despite the comprehensive evidence summarized, several limitations must be acknowledged. Substantial methodological heterogeneity, including differences in inclusion criteria, sampling strategies and measurement instruments used to assess alcohol consumption, knowledge of FASDs, and risk perception, complicates direct comparisons and restricts the generalizability of the results. Furthermore, the reliance on small, non-representative samples in many studies further limits the external validity of the evidence.

The predominant use of self-reported measures to capture alcohol consumption, attitudes, and knowledge introduces additional bias, including social desirability, particularly in contexts where prenatal alcohol use is stigmatized. These factors may underestimate both actual consumption and permissive attitudes, compromising data accuracy.

Taken together, these limitations underscore the urgent need for future research to adopt rigorous, standardized methodological frameworks and to implement validated, internationally comparable instruments, complemented by objective measures. Such approaches are essential to generate robust, high-quality evidence that accurately characterizes gestational alcohol exposure and clarifies its associated risks, thereby informing effective prevention and intervention strategies. Strengthening the evidence base in this way is crucial not only for advancing scientific understanding but also for informing evidence-based prevention programs, shaping public health policy, and guiding targeted interventions aimed at reducing prenatal alcohol exposure and its long-term consequences.

## Figures and Tables

**Table 1:** Analysis of the methodological quality of the articles.

	Total Score	Q	I1–I4–I6–I7	I2	I3	I5	I8
Addila et al. (2021) [28]	7	high	+	+	+	+	?
Balachova et al. (2016) [29]	7	high	+	+	+	+	?
Caires & Santos (2020) [30]	6	moderate	+	−	+	+	−
Chambers et al. (2005) [31]	6	moderate	+	?	+	+	?
Cornelius et al. (1997) [32]	7	high	+	+	+	+	?
Corrales-Gutierrez et al. (2019) [33]	8	high	+	+	+	+	+
Dumas et al. (2018) [34]	8	high	+	+	+	+	+
Eaton et al. (2014) [35]	7	high	+	+	+	+	−
Franco et al. (2020) [36]	6	moderate	+	?	+	+	?
Hen-Herbst et al. (2021) [37]	6	moderate	+	?	+	+	?
Howlett et al. (2017) [38]	7	high	+	+	+	+	?
Huang et al., 2023 [39]	6	moderate	+	?	+	−	+
Kaskutas (2000) [40]	7	high	+	+	+	+	?
Kristjanson et al. (2007) [41]	6	moderate	+	+	+	−	?
Kim & Park (2011) [42]	5	moderate	+	−	+	−	−
Lee et al. (2010) [43]	6	moderate	+	+	+	−	?
Lelong et al. (1995) [44]	7	high	+	+	+	+	?
Louw et al. (2018) [45]	6	moderate	+	−	+	+	?
McKnight & Merret (1987) [46]	6	moderate	+	+	+	−	?
Oechsle et al. (2020) [47]	6	moderate	+	+	+	−	?
Onwuka et al. (2016) [48]	5	moderate	+	+	−	−	?
Piotrkowicz et al. (2023) [49]	5	moderate	+	?	+	−	?
Senecky et al. (2011) [50]	6	moderate	+	?	+	+	?
Testa & Riefman (1996) [51]	5	moderate	+	?	+	−	−
Ujhelyi et al. (2022) [52]	7	high	+	+	+	+	?
Watt et al. (2016) [53]	7	high	+	+	+	+	?
West et al., (2024) [54]	8	high	+	+	+	+	+
Zabotka et al. (2017) [55]	4	moderate	+	−	?	−	−
Zahumensky et al. (2024) [56]	5	moderate	+	?	+	−	−

Q: Quality; I1: Appropriate approach: understanding people’s opinions, points of view and/or experiences in relation to a specific context/circumstance, I2: Sampling strategy: selection of participants/environments (the full range of relevant experiences and/or variables has been considered), I3: Data collection, I4: Data analysis, I5: Researcher’s position: relationship/perspective/experience of the authors with the research question, I6: Results, I7: Conclusions, I8: Findings’ transference; +: Yes, −: No, ?: Unclear.

## Data Availability

Data sharing is not applicable to this article as no new data were created or analyzed in this study.

## Abbreviations

The following abbreviations are used in this manuscript:

AEP	Alcohol-Exposed Pregnancy
ARCMs	Alcohol-Related Congenital Malformations
ARND	Alcohol-Related Neurodevelopmental Disorder
ARSMA-II	Acculturation Rating Scale for Mexican Americans-II
AUDIT	Alcohol Use Disorders Identification Test
AUDIT-C	Alcohol Use Disorders Identification Test–Consumption
BD	Binge Drinking
CEBM	Centre for Evidence-Based Medicine
DrInc	Drinker’s Inventory of Consequences
EDADES	Survey on Alcohol and Drugs in Spain (Encuesta sobre Alcohol y Drogas en España)
EPDS	Edinburgh Postnatal Depression Scale
FAE	Fetal Alcohol Effects
FAS	Fetal Alcohol Syndrome
FASDs	Fetal Alcohol Spectrum Disorders
GAC	Gestational Alcohol Consumption
GAD-2	Generalized Anxiety Disorder-2
HED	Heavy Episodic Drinking
I1–I8	Quality assessment items (Table 1)
IQ	Intelligence Quotient
OB/GYN	Obstetrics and Gynecology
OH	Alcohol
OSSS-3	Oslo 3-item Social Support Scale
pFAS	Partial Fetal Alcohol Syndrome
PAE	Prenatal Alcohol Exposure
PHQ-2	Patient Health Questionnaire-2
PHQ-9	Patient Health Questionnaire-9
PRISMA	Preferred Reporting Items for Systematic Reviews and Meta-Analyses
Q	Quality Score (Table 1)
SAQ	Student Alcohol Questionnaire
SD	Standard Deviation
T-ACE	Tolerance–Annoyed–Cut down–Eye-opener questionnaire
TWEAK	Tolerance–Worry–Eye-opener–Amnesia–Kut down questionnaire
V/I	Variables/Instruments
WOS	Web of Science
